# Influence of HCl Concentration on Corrosion Behavior between Au or Cu Bonding Wires and the Bond Pad for Semiconductor Packaging

**DOI:** 10.3390/ma16237275

**Published:** 2023-11-22

**Authors:** Young-Ran Yoo, Gyubinn Kim, Sung-Min Jeon, Hyun-Jun Park, Won-Wook Seo, Jeong-Tak Moon, Young-Sik Kim

**Affiliations:** 1 Materials Research Centre for Energy and Clean Technology, Andong National University, 1375 Gyeongdong-ro, Andong 36729, Republic of Korea; 2 Department of Materials Science and Engineering, Andong National University, 1375 Gyeongdong-ro, Andong 36729, Republic of Korea; 20225049@student.anu.ac.kr; 3 MK Electron Co., Ltd., 405 Geumeo-ro, Pogok, Yongin 17030, Republic of Korea

**Keywords:** semiconductor, bonding wires, Al bond pad, galvanic corrosion, HCl concentration

## Abstract

Wire bonding, one of the methods for electrically connecting a semiconductor chip with a substrate, involves attaching thin metal wires to pads. It is the oldest electrical connection method that exhibits high compatibility with other processes. The metal wires used for electrical connection in wire bonding are mainly made of Au, Cu, and Ag. After the wire bonding, molding is performed using the epoxy molding compound (EMC). However, EMC inevitably contains ions such as halogen elements. In addition, it absorbs moisture due to its hydrophilicity, creating a corrosive environment with electrolytes. In this study, we evaluated the influence of hydrochloric acid concentration on corrosion behavior between Au or Cu bonding wires and sputtered Al bond pads. The electrochemical factors such as corrosion potential difference (ΔE), galvanic corrosion current density (i_g_), and anodic and cathodic Tafel slopes were found to influence galvanic corrosion behavior. Galvanic corrosion tendency in first bond and second bond areas of PCB unit specimen was confirmed.

## 1. Introduction

Semiconductor chips go through the packaging process, which entails creating a mechanism for them to send and receive signals and make them safe and protected from various environmental factors [1]. There are several ways to electrically connect the semiconductor chips and substrates, including wire bonding, flip chip bonding, and through silicon [2,3,4,5].

Wire Bonding is a method that bonds a thin metal wire to the pad to create an electrical connection. Compared to the bump used in flip chip bonding, the wire is much longer and has a small diameter, which increases the time to transmit electrical signals and requires space for wire formation; however, it has flexible characteristics, and it is easy to adjust the bonding position [6]. In addition, as the oldest electrical connection method [7,8], the technology is mature and has the advantages of good compatibility with other processes and low cost; therefore, it is used in parallel with other processes [8,9]. The types of wires used for wire bonding are mainly Au, Ag, and Cu. The wire used for wire bonding has a fine thickness and directly affects the efficiency and reliability of the semiconductor module. Therefore, it is important to use a wire with excellent corrosion resistance and joint degradation characteristics.

Au wire is characterized by good current flow, chemical stability, good corrosion resistance and conductivity, and good electrical conductivity, making it suitable for producing small-diameter wires [1,10,11]. It is most used in the packaging industry due to its excellent bonding properties by forming Al pads and intermetallic compound (IMC) quickly, but due to its high price, alternative materials have been used [1,3,12,13,14,15,16,17]. However, it is still the main bonding wire for products with sensitive bond pads. Cu wire requires different process conditions than Au wire, which increases the process costs [7]. In addition, its hardness makes it difficult to form balls and necessitates high pressure during bonding, which can cause cracks in the pad [1]. However, Cu is easily oxidized upon exposure to air due to its low corrosion resistance [1,10,18,19]. Therefore, to increase its lifespan, it is often alloyed or coated with materials that have good corrosion resistance (such as Pd and Au) [1]. Compared to Au wire, the cost of substrate materials is low [1,12,20,21,22]. In addition, it has the advantages of high electrical conductivity and tensile strength, low thermal resistance, and low electrical resistance [1,7,8,12,13,14,17,20,22]. Therefore, it is widely used in nanoelectronics packaging [23].

After the electrical connection is made by bonding the various types of wires on the Al bond pad, the semiconductor chip is sealed to protect it from environmental factors such as heat, shock, and moisture through the encapsulation process. The encapsulation process mainly involves the use of epoxy molding compound (EMC). EMC is widely applied for semiconductor packaging due to its low cost and easy processability, but it inevitably contains halogen elements and ions such as sulfur [8,10,13,20,24,25,26]. Due to the hydrophilic nature of EMC, it readily absorbs moisture from the air and promotes ion diffusion, creating a corrosive environment with electrolytes [6,20,27,28]. In particular, chloride (Cl^−^) ions can weaken or dissolve passivation films in humid environments, causing corrosion, accelerating oxidation-reduction reactions, and intermetallic compound (IMC) formation, which is closely related to the failure mechanism of wire bonds [8,13,21,29].

In corrosive environments, galvanic corrosion occurs in bonding wires and Al bond pads bonded together due to the potential differences [8,13,17]. The bonding wire with a high potential has corrosion resistance, while the Al bond pad with a low potential corrodes, causing the formation of cracks [6,17,30,31,32,33]. As these cracks propagate to the center of the bond, they accelerate the crack growth due to crevice corrosion, causing the separation of the wire and pad [8,13,26]. This separation depends on the growth rate and characteristics of the IMC generated between the wire and the Al bond pad [5,34]. The IMC is formed by mutual diffusion between the ball and the Al pad at the temperature generated during the bonding process [26]. A moderate amount of IMC growth improves the bond between the wire and Al. However, excessive IMC growth can increase the contact resistance, worsen the heat generation at the interface, cause brittleness, and lead to short circuit of the wire [7,35].

Au wire generally forms IMCs with Al pads quickly and densely and has excellent bonding properties, but it has a large potential difference with Al pads [7,36]. Nevertheless, IMC prevents the intrusion of halogen elements and moisture, resulting in a low corrosion rate and a delayed delamination time [17]. In the case of Cu wire, the formation of IMC with Al pad is slower [11,12], the bonding properties are poor, and it is easy to produce a thin oxide layer, but the thickness of the oxide continues to increase over time [3,7,37], so it may fail faster than Au wire [17]. However, Cu-Al intermetallic compounds are more reliable at high temperatures than Au wires due to less void formation [22]. IMC formed between the Cu wire and the Al pad has a potential between Cu and Al, and the passivation film is not stable, forming a sacrificial anode at the interface of the wire and Al, which is preferentially corroded [5,6,13,38].

In a wire bonding process, the wire bond is comprised of a first bond made on the die bonding pad and a second bond made on the substrate bonding pad. Typically, the first bond is referred to as a ball bond, while the second bond is known as a stitch bond. Both bonds are created on the PCB substrate, with the stitch bond being smaller than the ball bond, making it more susceptible to issues related to the plating quality and surface contamination on the pads, which may affect its adhesion [12].

As discussed above, several studies have investigated the corrosion behavior of the wire and bond interface according to the concentration of these halogen ions (especially Cl^−^ ions). However, there is a paucity of research on the galvanic corrosion behavior of the original materials of wire and the bond pad. Therefore, in this study, we evaluated the effect of hydrochloric acid (HCl) concentration on the galvanic corrosion behavior of the bond pad with galvanic coupled Au wire and Cu wire and analyzed the electrochemical factors that affect the galvanic corrosion behavior. The Galvanic corrosion test of first bond and second bond on PCB unit specimen was performed at 85 °C and 85% relative humidity and analyzed.

## 2. Materials and Methods

### 2.1. Test Specimen

In this study, the Al bond pad, solid Al, and bonding wire were used for testing. Solid Al specimen was used for the comparison with sputtered Al specimen and the commercial wrought pure Al plate. The Al Bond Pad was fabricated by depositing Al on a 4-inch Si wafer using a DC Magnetron Sputter (Korea Vacuum Tech, KVS-2002L, Gimpo, Republic of Korea). Then, 99.999% purity was used as the Al target, and 605 nm deposition was achieved using a pressure of 1 mTorr and a power of 50 W. The sputtering process was followed by electrochemical tests. For subsequent electrochemical testing, the sputtered wafers were diced and connected to copper wires using carbon tape. The electrically connected specimens were insulated using acid-resistant epoxy except for the size of 0.04 cm^2^.

Bonding wire is made from refined materials that have been purified by excluding impurities and then melted and alloyed to determine the type of wire. The alloyed wire is produced by continuous casting to produce the initial wire, which is then machined to reduce the diameter, followed by heat treatment and winding. The bonding wire used in this study was provided by MK Electron Co., Ltd. (Yongin, Republic of Korea), and used 99.99% Au and 99.99% Cu with a diameter of 25 μm.

**Figure 1:**
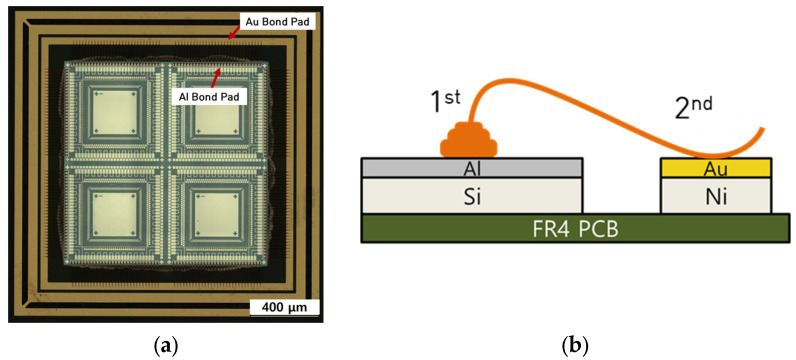
(**a**) Wire-bond unit specimen, (**b**) Schematic diagram on 1st bond and 2nd bond areas.

Wire-bond unit specimen was manufactured using wire bonder of MK electronics Co. Figure 1 shows the unit specimen and schematic diagram on 1st bond and 2nd bond areas. Wire bonding is performing using a ball bonder (K&S RAPID PRO, Kulicke & Soffa, Singapore) and Table 1 shows the bond parameters. The thickness of the sputtered Al layer on unit specimen was about 10,000~12,000 Å.

### 2.2. Polarization Test

Polarization tests were performed to evaluate the corrosion properties of the Al bond pad and bonding wire. To evaluate the behavior according to HCl concentration, solutions of 1% NaCl, 1% NaCl + 0.01% HCl, 1% NaCl + 0.1% HCl, and 1% NaCl + 1% HCl at 25 °C were used after deaeration for 30 min at the rate of 200 mL/min using N_2_ gas. The test equipment was a Potentiostat (Interface 1000, Gamry, Warminster, PA, USA). A saturated calomel electrode (SCE) was used as the reference electrode, and a platinum electrode was used as the counter electrode. After 60 s of conditioning and 150 s of initial delay, the corrosion potential progressed from −0.7 V to 1 V (SCE) at a scan rate of 0.33 mV/s.

### 2.3. Electrochemical Galvanic Corrosion Test

Electrochemical galvanic corrosion tests were conducted to evaluate the galvanic corrosion behavior of Al bond pads with galvanic coupled bonding wires. A SCE was used as the reference electrode, the Al pad was the working electrode, and the bonding wire served as the countering electrode. The test solution was identical to that used for polarization behavior evaluation, and to eliminate the influence of surface area, the ratio of anodic and cathodic areas was set to 1:1.

### 2.4. Temperature Humidity Test (THT)

**Figure 2:**
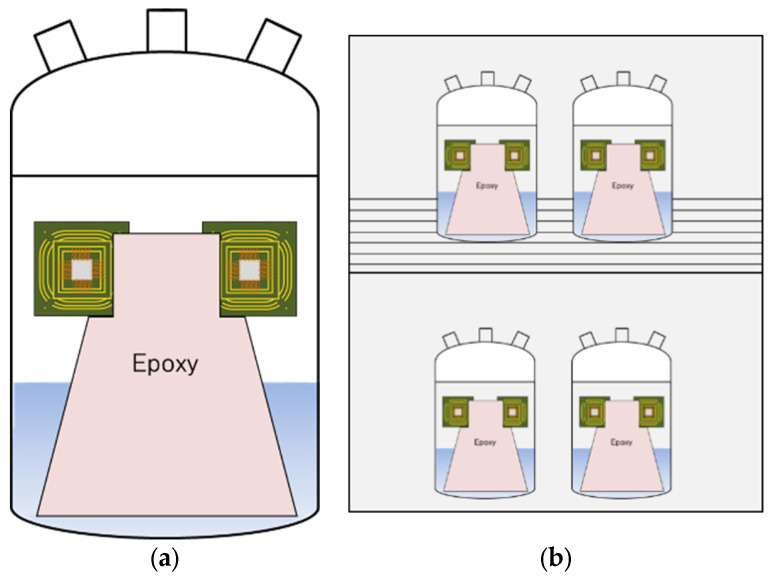
Corrosion cell in the chamber of Temperature-Humidity Test; (**a**) schematic of a unit cell installation in a glass cell, (**b**) schematic of glass cell installed in THT chamber.

A temperature humidity test was performed for 100 h using the controlled chamber (PR-2J, ESPEC, Osaka, Japan). The unit specimen was fixed on the epoxy mold and the mold was placed in the test solution of the glass cell a shown in Figure 2a, and 4 cells were installed in THT chamber like in Figure 2b. Test solutions were 1% NaCl and 1% NaCl + 0.1% HCl at 85 °C and 85% R.H.

### 2.5. Optical and Three-Dimensional Microscopic Observation and SEM-EDS Analysis

The morphology of specimens was assessed before and after testing using an Optical Microscope (OM, AXIOTECH 100 HD, ZEISS, Oberkochen, Germany). The surface profile was measured at ×100 magnification using a three-dimensional (3D) microscope (VK-X3000, Keyence, Japan). Field Emission Scanning Electron Microscope (FE-SEM, MIRA3XMH, Tescan, Brno, Czech Republic). The elemental distribution was analyzed using EDS (Energy Dispersive X-ray Spectrometer, Mmax 50, Oxford, UK).

### 2.6. X-ray Diffraction (XRD) Analysis

The solid Al was polished using SiC paper up to #2000 and the sputtered Al was analyzed as-sputtered state. XRD analysis on the solid Al and sputtered Al was performed using the X-ray Diffractometer (Ultima IV, Rigaku, Tokyo, Japan) and 2θ was diffracted from 5° to 85° at a rate of 4°/min.

## 3. Results and Discussion

### 3.1. Corrosion Behavior of Single Specimen

**Figure 3:**
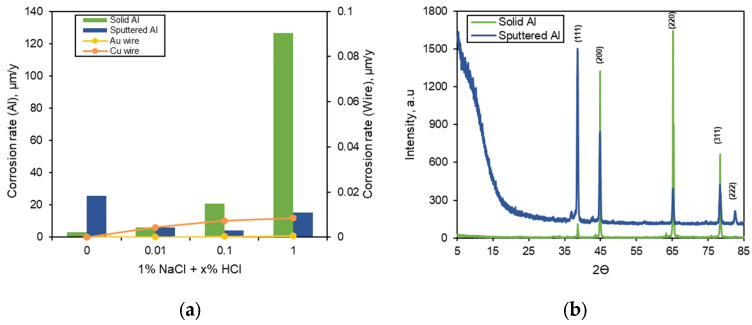
(**a**) Effect of HCl concentration on the corrosion rate of solid Al, sputtered Al, and bonding wires as a single specimen in deaerated 1% NaCl + x% HCl solution at 25 °C and (**b**) XRD patterns of solid Al and sputtered Al specimens.

Figure 3a shows the effect of HCl concentration on the corrosion rate of solid Al, sputtered Al, and bonding wires as a single specimen by polarization test in deaerated 1% NaCl + x% HCl solution at 25 °C. Solid Al generally exhibits weak corrosion resistance in environments where Cl^−^ ions are present [39]. In the case of solid Al, the corrosion rate increased significantly with the increase in the concentration of HCl. However, an increase in HCl concentration did not significantly affect the corrosion resistance of sputtered Al used in the bonding pad. The corrosion rates of Au and Cu wires used as bonding wire materials tended to increase with the increase in HCl concentration, but there was a minimal effect on the corrosion rate. Among the four specimens, the Au wire showed the lowest corrosion rate. Solid Al showed a high corrosion rate depending on the HCl concentration, but sputtered Al showed a relatively small value. Table 2 shows the corrosion current density and corrosion potential of the test specimens by polarization curves.

For sputtered Al, the corrosion rate varied with the HCl concentration, but the effect was very small. This difference in corrosion rate compared to solid Al was due to the crystal structure of Al. Figure 3b shows the XRD patterns of solid Al and sputtered Al specimens. The sputtered Al exhibits some amorphous structure, which leads to a difference in corrosion behavior compared to solid Al. It has been reported that Al alloy with an amorphous structure forms a passive film in a chloride-containing solution and has excellent corrosion resistance by increasing the pitting potential [40,41].

**Figure 4:**
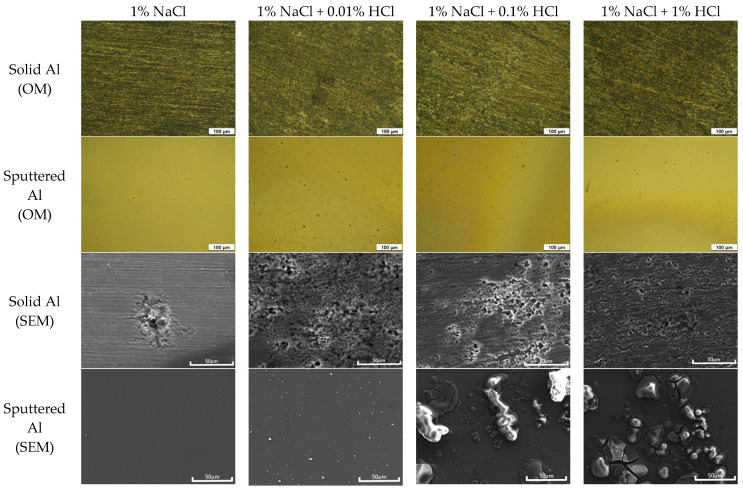
Surface morphology of solid Al and sputtered Al after polarization tests in deaerated 1% NaCl + x% HCl solution at 25 °C.

After the polarization test, the changes in the surface morphology of Al specimens were observed by optical microscope (OM) and scanning electron microscope (SEM). Figure 4 shows the surface morphology of solid Al and sputtered Al after polarization tests in deaerated 1% NaCl + x% HCl solution at 25 °C. In the optical image, it can be seen that the overall corrosion of solid Al increased with the increase in the concentration of HCl, and there was a progression of localized corrosion. However, in the case of sputtered Al, the patterns formed did not exhibit significant differences even with the increase in the acid concentration. As shown in the SEM images, in the case of solid Al, localized corrosion was accelerated, while the sputtered Al showed the formation of corrosion products on the surface. The lack of a significant difference in corrosion rate with an increase in HCl concentration is likely attributable to the formation of these corrosion products.

### 3.2. Galvanic Corrosion Behavior between Au or Cu Wires and Al Pad

**Figure 5:**
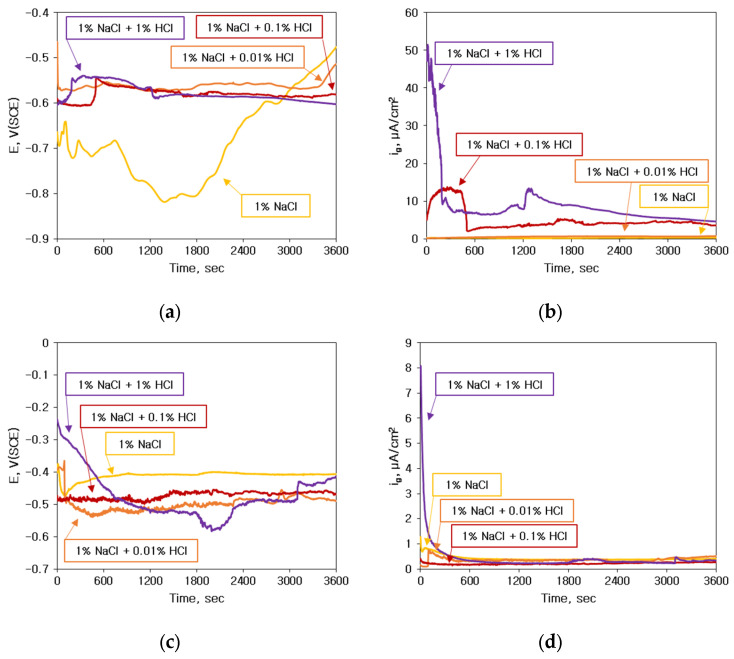
Effect of HCl concentration on the electrochemical galvanic corrosion between bonding wires and Al bond pad; (**a**) Galvanic corrosion potential and (**b**) Galvanic current density between Au wire and sputtered Al, and (**c**) Galvanic corrosion potential and (**d**) Galvanic current density between Cu wire and sputtered Al.

When various types of bonding wires are electrically connected by bonding with Al bond pads, and a corrosive environment containing Cl^−^ ions is formed around them, the potential difference between the wire and Al induces galvanic corrosion. Therefore, to investigate the tendency of galvanic corrosion according to HCl concentration, Au and Cu wires were connected as a galvanic couple with sputtered Al, and an electrochemical galvanic corrosion test was performed. Then the relatively active Al was connected to the working electrode, and the bonding wire was connected to the counter electrode to observe the galvanic corrosion current density and galvanic corrosion potential. Figure 5 shows the effect of HCl concentration on the electrochemical galvanic corrosion between bonding wires and sputtered Al bond pad. Figure 5a,b show the galvanic corrosion potential and galvanic current density between Au wire and Al. The galvanic current density tended to increase with the increase in HCl concentration. The effect of HCl concentration on the electrochemical galvanic corrosion potential and Galvanic current density between Cu wire and sputtered Al is shown in Figure 5c,d. The change in HCl concentration showed no distinct effect on the galvanic corrosion current density between Cu wire and Al.

**Figure 6:**
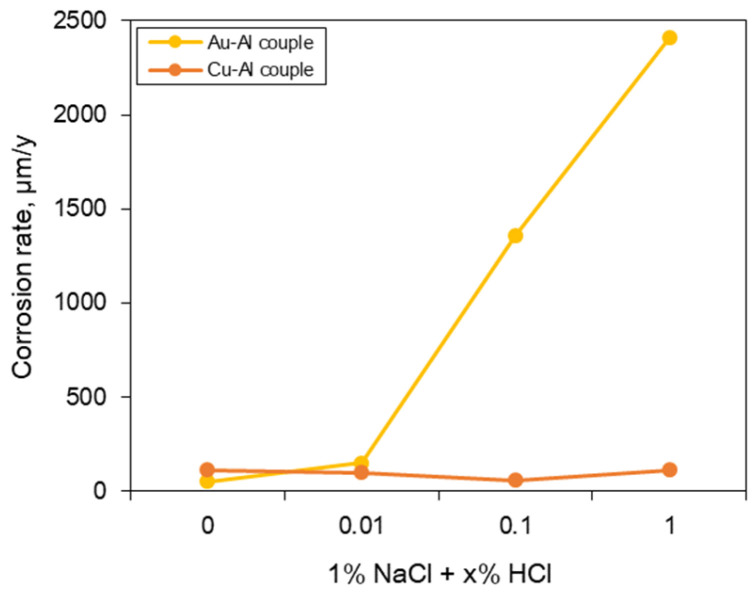
Effect of HCl concentration on the galvanic corrosion rate of sputtered Al specimen coupled with Au or Cu wires in deaerated 1% NaCl + x% HCl at 25 °C.

Figure 6 shows the effect of HCl concentration on the galvanic corrosion rate of sputtered Al specimen coupled with Au or Cu wires in deaerated 1% NaCl + x% HCl at 25 °C. The corrosion rate when Au wire and Al were connected in a galvanic coupled was higher than that of the single sputtered Al specimen upon exposure to the corrosive environment. The corrosion rate increased with the increase in the HCl concentration. On the other hand, when Cu wire and Al were connected in a galvanic couple, the corrosion rate of Al did not change significantly despite the increase in HCl concentration. This difference in corrosion behavior was analyzed by observing the surface of Al after the galvanic corrosion test.

**Figure 7:**
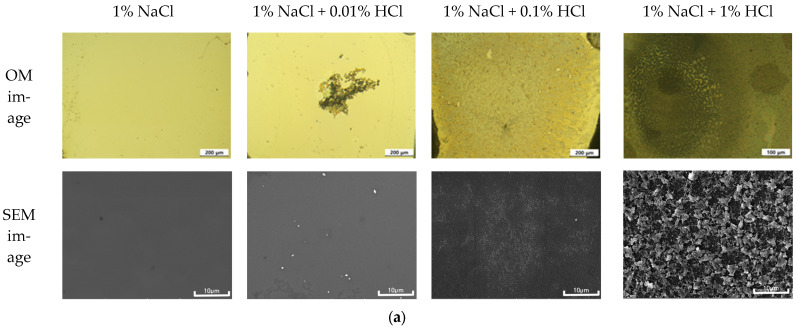

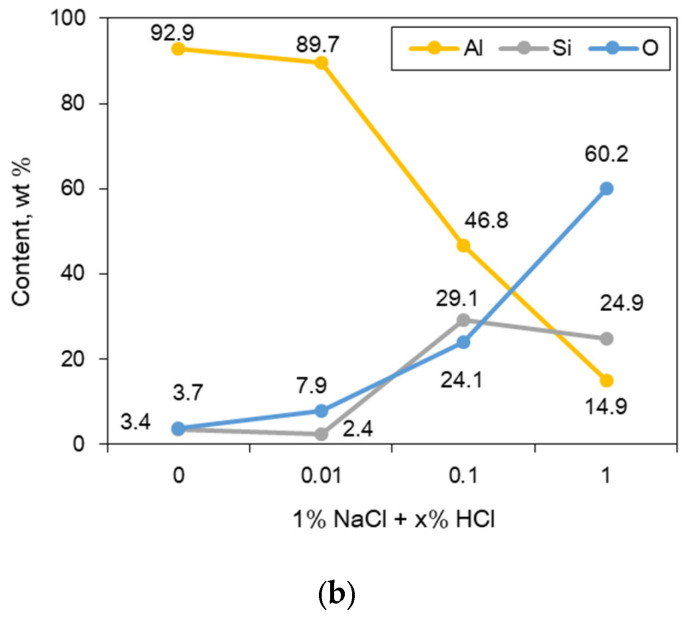
(**a**) Surface morphology of sputtered Al specimen after galvanic corrosion test with Au wire in deaerated 1% NaCl + x% HCl at 25 °C and (**b**) elemental distribution by EDS analysis on the surface after galvanic corrosion test.

Figure 7a shows the surface morphology of the sputtered Al specimen after galvanic corrosion test with Au wire in deaerated 1% NaCl + x% HCl at 25 °C. It can be seen that the corrosion of the Al surface accelerated with the increase in the concentration of HCl. The SEM image analysis showed an increase in the surface corrosion products with the increase in HCl concentration. Figure 7b shows the elemental distribution by EDS analysis on the surface after the galvanic corrosion test. Surface analysis of Al, Si, and O demonstrated that as the concentration of HCl increased, Al was locally dissolved, and oxides were formed. These corrosion products were removed from the surface as the corrosion environment became more severe, reducing the amount of Al and exposing Si. In particular, at 1% HCl concentration, the corrosion rate increased due to the rapid dissolution of both Al and Si.

**Figure 8:**
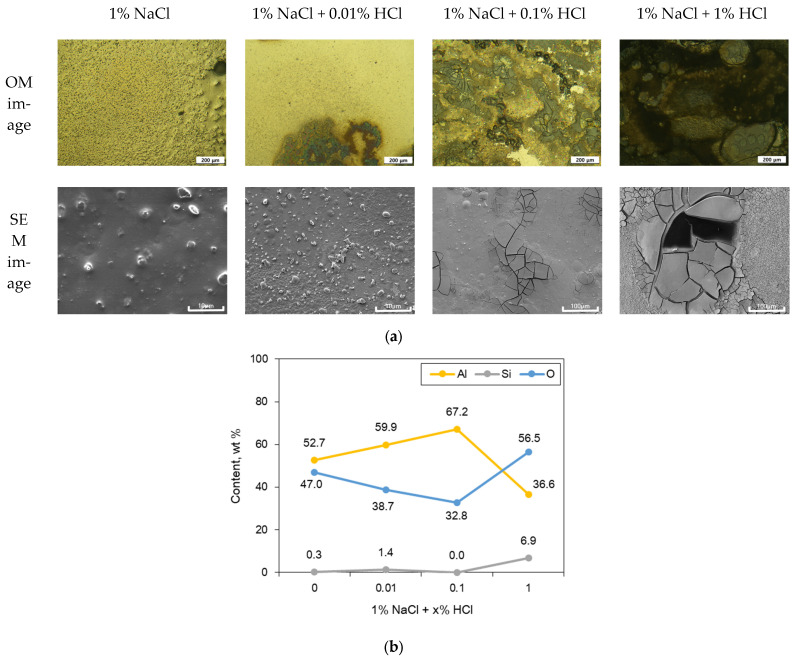
(**a**) Surface morphology of sputtered Al specimen after galvanic corrosion test with Cu wire in deaerated 1% NaCl + x% HCl at 25 °C and (**b**) elemental distribution by EDS analysis on the surface after galvanic corrosion test.

A slightly different trend was observed in the galvanic couple of Cu wire and Al. Figure 8a shows the surface morphology of the sputtered Al specimen after galvanic corrosion test with Cu wire in deaerated 1% NaCl + x% HCl at 25 °C. As the concentration of HCl increased, the corrosion products on the Al surface tended to increase. The oxide formed on the surface was partially cracked at a concentration of 0.1% HCl. At 1% HCl concentration, the cracks became denser and some of the Al oxide films delaminated, exposing the Si. The corrosion products on the Al surface tended to increase with the increase in HCl concentration. The oxides formed on the surface partially showed the shape of cracks at an HCl concentration of 0.1%. At 1% HCl concentration, the cracks became denser and, in some cases, delamination of the Al oxide film occurred, exposing the Si. These results can also be seen in the surface analysis graph. Figure 8b shows the elemental distribution by EDS analysis on the surface after the galvanic corrosion test. The Al content increased with the increase in HCl concentration up to 0.1%, but at 1% HCl concentration, O and Si increased, and Al decreased. This was because of the delamination of Al oxide and exposure of Si. Exposure of Si of the matrix structure accelerates the galvanic corrosion rate with the dissolution of Al.

In the galvanic coupled between Au wire and Al, Al forms an oxide but dissolves as the concentration of HCl increases and less remains on the surface. Otherwise, in the galvanic coupled between Cu wire and Al, Al corrodes as the concentration of HCl increases but remains on the surface as Al oxide without causing a significant change in the galvanic corrosion rate.

**Figure 9:**
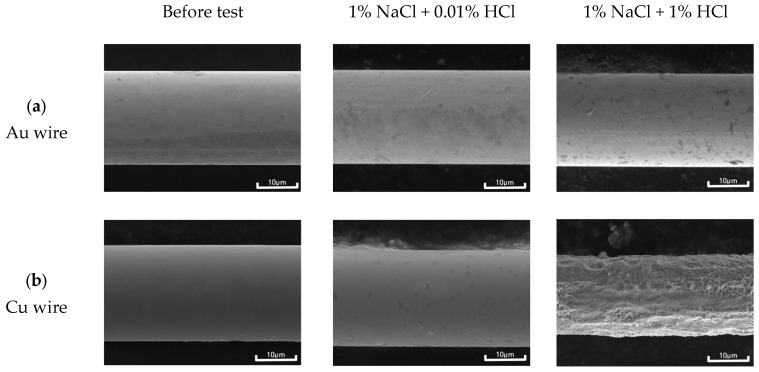
Surface morphology of (**a**) Au wire and (**b**) Cu wire before and after the galvanic corrosion test with sputtered Al pad in deaerated 1% NaCl + x% HCl solution at 25 °C.

Generally, when dissimilar metals are connected in a corrosive environment, galvanic corrosion occurs wherein the active metal accelerates corrosion, and the noble metal is protected. Al is a relatively active material for galvanically coupled bonding wires. The galvanic current density results showed positive current density values for active Al. Therefore, the bonding wire should be protected by the sacrificial anode effect of Al. Figure 9 shows the surface morphology of (a) Au wire and (b) Cu wire before and after the galvanic corrosion test with sputtered Al pad in deaerated 1% NaCl + x% HCl solution at 25 °C. While Au wire showed some formation of corrosion products on its surface as the concentration of HCl increased, it did not show any significant changes compared to the surface before the test. However, in the case of Cu wire, the surface morphology changed as the concentration of HCl increased, causing the formation of corrosion products and partial dissolution. In particular, with a further increase in the HCl concentration to 1%, the dissolution of Cu wire resulted in a reduction in wire thickness. When forming a galvanic coupled with Al, the Cu wire is protected by the sacrificial anode effect of Al in a mildly corrosive environment. However, in a severely corrosive environment, both Al and Cu wires are corroded and cannot be completely protected by Al. Therefore, the corrosion rate of Al in the galvanic coupled between Cu wire and Al is not obvious when evaluating the effect of HCl concentration.

**Figure 10:**
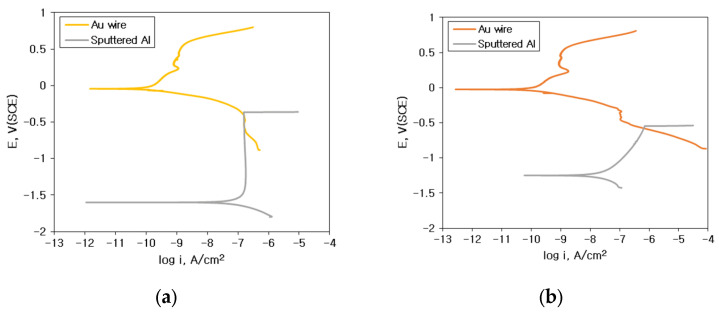

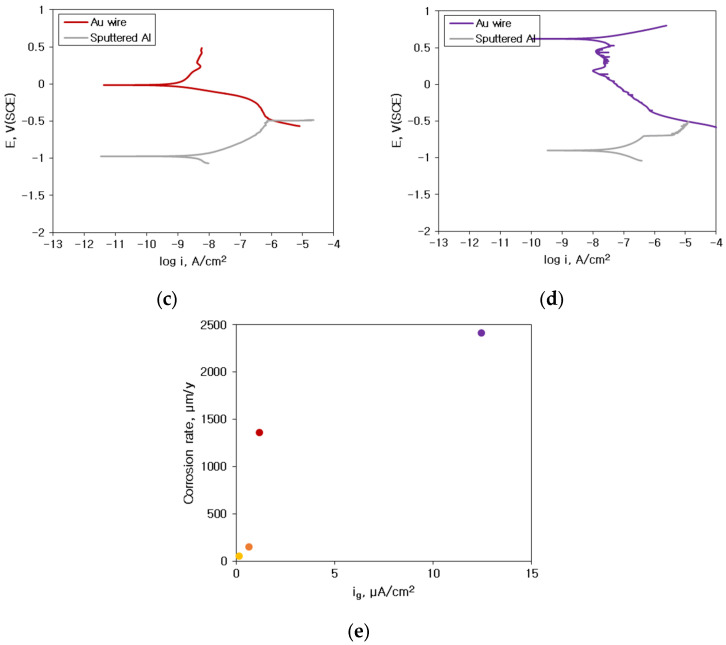
Analysis of galvanic corrosion using the mixed potential theory between sputtered Al specimen and Au wire: (**a**) 1% NaCl, (**b**) 1% NaCl + 0.01% HCl, (**c**) 1% NaCl + 0.1% HCl, (**d**) 1% NaCl + 1% HCl, and (**e**) relationship between corrosion rate obtained by galvanic test and galvanic current density (i_g_) estimated by the mixed potential theory.

**Figure 11:**
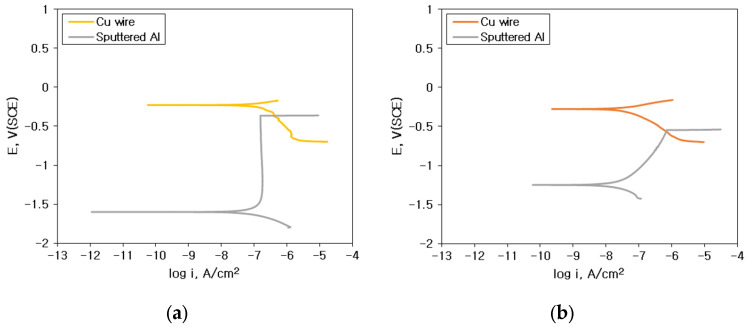

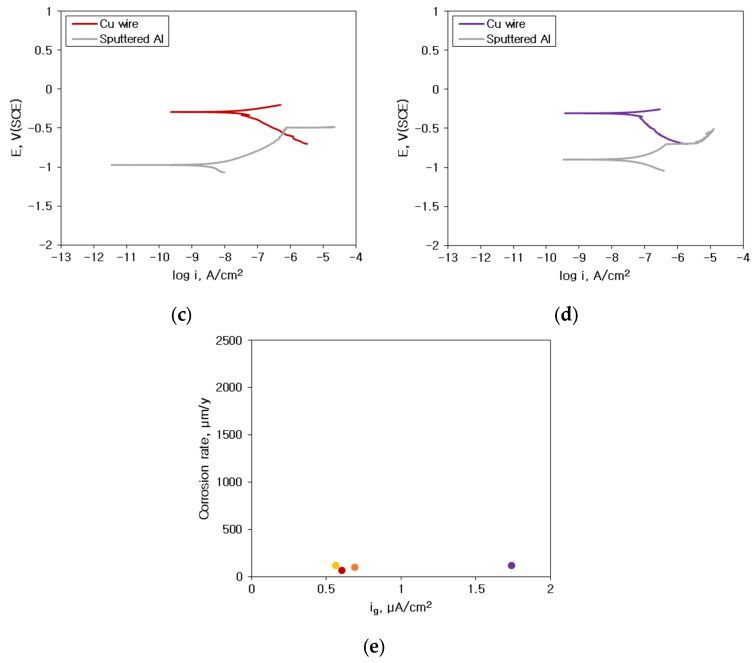
Analysis of galvanic corrosion using the mixed potential theory between sputtered Al specimen and Cu wire: (**a**) 1% NaCl, (**b**) 1% NaCl + 0.01% HCl, (**c**) 1% NaCl + 0.1% HCl, (**d**) 1% NaCl + 1% HCl, and (**e**) relationship between corrosion rate obtained by galvanic test and galvanic current density (i_g_) estimated by the mixed potential theory.

Galvanic corrosion behavior can be predicted by mixed potential theory. Figure 10 and Figure 11 show the analysis of galvanic corrosion using the mixed potential theory between sputtered Al specimen and Au wire: (a) 1% NaCl; (b) 1% NaCl + 0.01% HCl; (c) 1% NaCl + 0.1% HCl; (d) 1% NaCl + 1% HCl. Based on the polarization behavior of these alloys, various electrochemical factors such as the corrosion potential difference (ΔE), galvanic corrosion current density (i_g_) by the mixed potential theory, anodic Tafel slope (β_A_), and cathodic Tafel slope (β_C_) can be obtained.

Figure 10e and Figure 11e show the relationship between the corrosion rate obtained by the galvanic test and galvanic current density (i_g_) estimated by the mixed potential theory (yellow dot: 1% NaCl, orange dot: 1% NaCl + 0.01% HCl, red dot: 1% NaCl + 0.1% HCl and purple dot: 1% NaCl + 1% HCl). In the case of galvanic couple with Au wire and Al, the galvanic corrosion rate increased with the increase in the i_g_. The i_g_ also showed a linear increase with the increase in HCl concentration. However, in the case of the galvanic couple with Cu wire and Al, the effect of i_g_ on the galvanic corrosion rate was less correlated.

**Figure 12:**
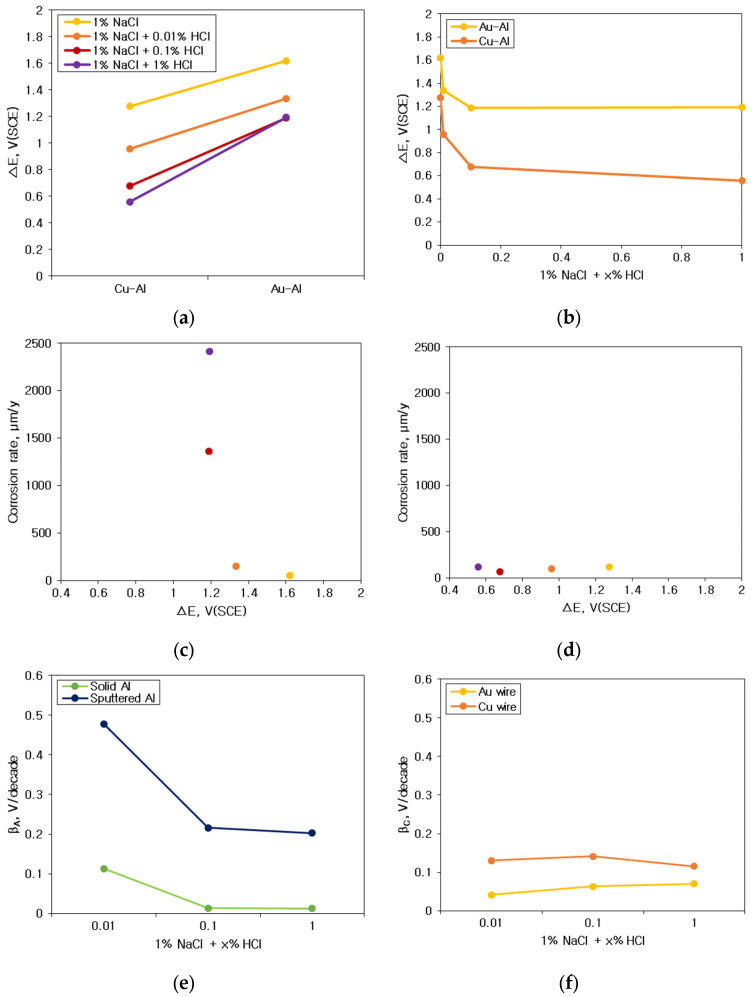
Interpretation of galvanic corrosion factors in deaerated 1% NaCl + x% HCl solution at 25 °C: (**a**) dissimilar metal vs. galvanic potential difference, (**b**) HCl concentration vs. galvanic potential difference, (**c**) corrosion potential difference vs. galvanic corrosion rate of Al with Au wire by HCl concentration, (**d**) corrosion potential difference vs. galvanic corrosion rate of Al with Cu wire by HCl concentration, (**e**) anodic Tafel slope by HCl concentration and (**f**) cathodic Tafel slope by HCl concentration.

**Figure 13:**
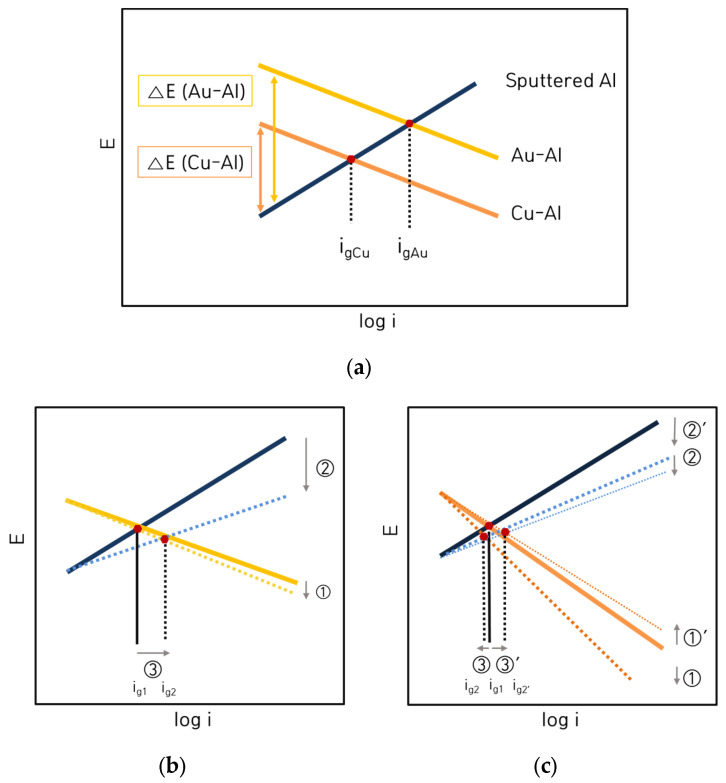
Galvanic corrosion models between bonding wires and Al bond pad; (**a**) dissimilar metal effect, (**b**) HCl concentration effect in Au wire—Al couple, (**c**) HCl concentration effect in Cu wire—Al couple.

Figure 12 shows the interpretation of galvanic corrosion factors in deaerated 1% NaCl + x% HCl solution at 25 °C. Figure 12a,b show the effect of ΔE on the galvanic corrosion behavior of two metals connected as a galvanic couple. As shown in Figure 12a, when the material connecting Al and the galvanic couple changed in the same environment, the ΔE between Au wire and sputtered Al, which showed a high corrosion rate with an increase in the concentration of HCl, became larger than those of Cu-Al couples. In other words, when the environment is constant, ΔE shows a high correlation with the galvanic corrosion rate. However, as shown in Figure 12b, for the same galvanic couple, when the HCl concentration increased, ΔE decreased despite the increase in galvanic corrosion rate. These findings indicate that for the same galvanic couple, the galvanic corrosion behavior with changing concentrations of HCl cannot be explained by ΔE. Figure 12c,d show the correlation of ΔE by mixed potential theory and galvanic corrosion rate (yellow dot: 1% NaCl, orange dot: 1% NaCl + 0.01% HCl, red dot: 1% NaCl + 0.1% HCl and purple dot: 1% NaCl + 1% HCl). Figure 12c shows the correlation between the ΔE by the mixed potential theory and the corrosion rate of Al with Au wire by HCl concentration. The ΔE decreased with an increase in the concentration of HCl. Figure 12d shows the correlation between the ΔE by the mixed potential theory and the corrosion rate of Al with Cu wire by HCl concentration. The correlation between HCl concentration and the ΔE was low. This indicates that the ΔE by the mixed potential theory has a low correlation with the galvanic corrosion rate. Figure 12e,f show the β_A_ β_C_ by polarization curves and galvanic corrosion behavior by HCl concentration. Figure 12e shows the β_A_ of Al with increasing HCl concentration in the polarization behavior. It shows that the β_A_ of solid Al and sputtered Al tended to decrease with an increase in the HCl concentration. Figure 12f shows a graph of the variation of β_C_ with the concentration of HCl by wire material. It shows that β_C_ increased for Au wires with an increase in the concentration of HCl but slightly decreased for Cu wires in 1% HCl solution. This comparison of β_A_ on Al and β_C_ on wire materials explains the effect of HCl concentration variation on galvanic corrosion behavior in the same galvanic couple, which is shown in Figure 13.

Figure 13 shows the galvanic corrosion models between bonding wires and Al bond pad. Figure 13a illustrates a model showing the dissimilar metal effect, which shows that the potential difference (ΔE) is the determinant of the galvanic corrosion behavior of dissimilar metals in the same environment. Figure 13b shows the HCl concentration effect in the Au wire—Al couple; the galvanic corrosion rate increased with the decrease in the β_A_ of Al (②) and the increase in the β_C_ of the Au wire (①). Figure 13c shows the HCl concentration effect in the Cu wire—Al couple, as the HCl concentration increased; the β_A_ of Al decreased (②, ②’), but the β_C_ of Cu wire increased (①) and then decreased again (①’). These findings indicate a close correlation of the β_A_ and the β_C_ with the galvanic corrosion rate.

### 3.3. Galvanic Corrosion Behavior in First and Second Bond Areas on the PCB

**Figure 14:**
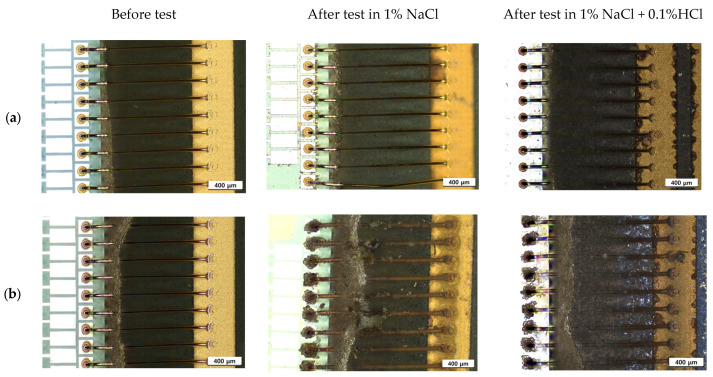
Surface appearance of unit specimen after ‘Temperature-Humidity Test’ at 85 °C and 85% relative humidity for 100 h; (**a**) Au wire bonded unit, (**b**) Cu wire bonded unit.

Figure 14 shows the surface appearance of PCB unit specimen after THT at 85 °C and 85% relative humidity for 100 h. In the case of corroded area in 1% NaCl, corrosion in Cu-wire bonded unit is more severe than that in Au wire bonded unit.

**Figure 15:**
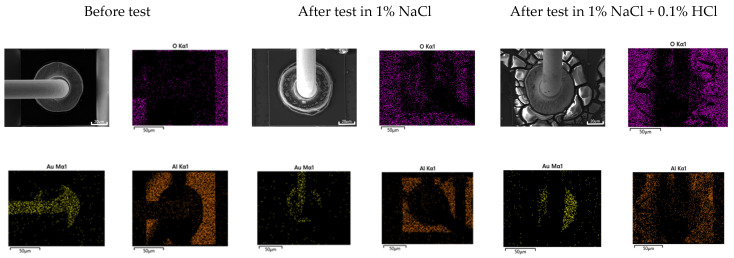
Elemental distribution of Au wire—Al pad 1st bond area before and after ‘Temperature-Humidity Test’ at 85 °C and 85% relative humidity for 100 h.

**Figure 16:**
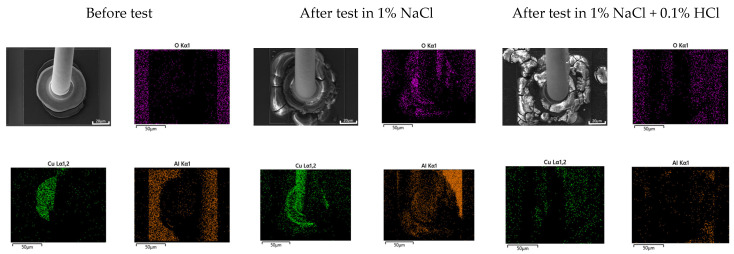
Elemental distribution of Cu wire—Al pad 1st bond area before and after ‘Temperature-Humidity Test’ at 85 °C and 85% relative humidity for 100 h.

Galvanic corrosion in the first ball bond area: Figure 15 shows the elemental distribution of the Au wire—Al pad first bond area before and after THT at 85 °C and 85% relative humidity for 100 h. The aluminum pad near the Au wire ball bond was oxidized, but the corrosion product was barely detected in the case of 1% NaCl. However, in the case of 1% NaCl + 0.1% HCl, the aluminum pad near the Au wire ball bond was severely corroded. On the other hand, Figure 16 shows the elemental distribution of the Cu wire—Al pad first bond area before and after THT at 85 °C and 85% relative humidity for 100 h. In the case of 1% NaCl and 1% NaCl + 0.1% HCl, the aluminum pad near Au wire ball bond was severely corroded.

Generally, galvanic corrosion between Au and Al will be more severe than that between Cu-Al. However, as shown in Figure 15, Au wire did passivate aluminum and oxidize aluminum and thus corrode less galvanically in 1% NaCl. This behavior can be explained using Figure 10a. Figure 10a shows that the cathodic curve of Au wire did encounter the passive state of Al pad. But, as shown in Figure 16, Cu wire did corrode aluminum in 1% NaCl. This behavior can be explained using Figure 11a. Figure 11a shows that the cathodic curve of Cu wire did encounter the passive state of the Al pad but did not passivate aluminum and facilitated corrosion of the Al pad.

**Figure 17:**
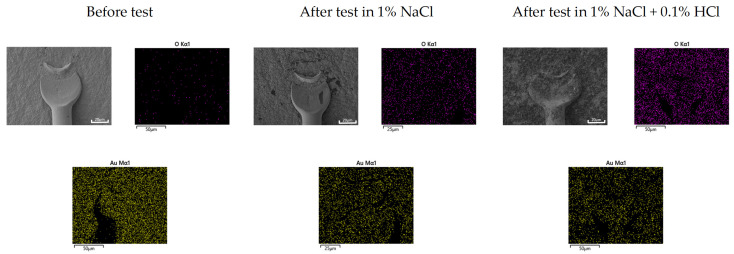
Elemental distribution of Au wire—Au pad 2nd bond area before and after ‘Temperature-Humidity Test’ at 85 °C and 85% relative humidity for 100 h.

**Figure 18:**
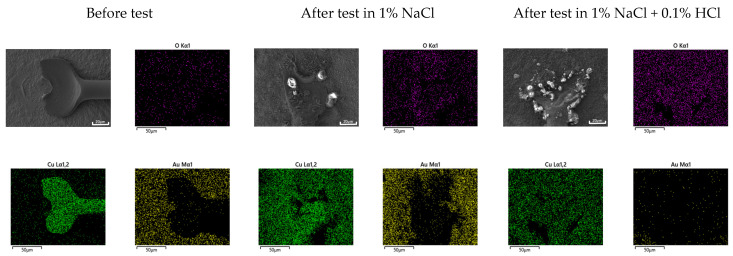
Elemental distribution of Cu wire—Au pad 2nd bond area before and after ‘Temperature-Humidity Test’ at 85 °C and 85% relative humidity for 100 h.

**Figure 19:**
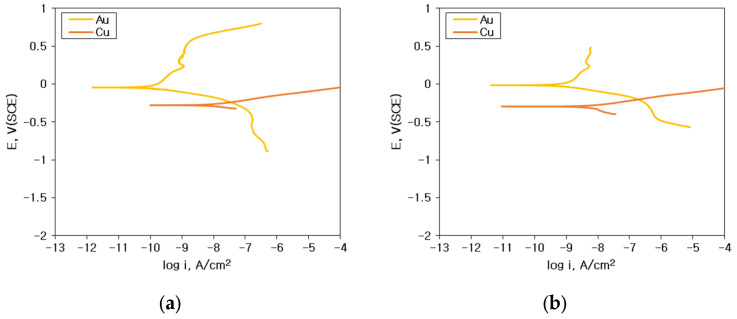
Analysis of galvanic corrosion in Cu wire—Au pad 2nd bond area using the mixed potential theory: (**a**) 1% NaCl, (**b**) 1% NaCl + 0.1% HCl.

Galvanic corrosion in second stitch bond area: Figure 17 shows the elemental distribution of the Au wire—Au pad second bond area before and after THT at 85 °C and 85% relative humidity for 100 h. Corrosive environments did corrode slightly Au wire and stitch bond areas. However, in the case of Cu wire stitch bond area, Cu wire dissolved and covered Au bond pad area as shown in Figure 18. This behavior can be explained using polarization curves. Figure 19 shows the polarization curves of Cu and Au wires in (a) 1% NaCl and (b) 1% NaCl + 0.1% HCl. The cathodic curves of the Au wire did encounter the active dissolution curve of the Cu wire. Therefore, copper oxide formed on the Au stitch bond area.

## 4. Conclusions

This study investigated the influence of electrochemical factors on the galvanic corrosion behavior between bonding wires and bond pad in 1% NaCl + x% HCl solution. The following conclusions were drawn:

1. The corrosion rate of the sputtered Al with HCl concentration was much lower than that of solid Al. This difference in corrosion rate is attributable to the presence of some amorphous structure in sputtered Al specimen. However, the corrosion rate of sputtered Al galvanically coupled with Au or Cu wires was greatly higher than that of single sputtered Al.
2. The galvanic corrosion rate of the galvanic coupled Al bond pad with Au or Cu wires was closely related to the potential difference in the same corrosion environment. On the other hand, the corrosion behavior of the same galvanic couples with different corrosion environments was affected by the anodic and cathodic Tafel slopes instead of the potential difference.
3. Galvanic corrosion tendency in the first bond and second bond areas of the PCB unit specimen depended upon the passive or transpassive or active states of the anodic material which encountered the cathodic materials in any corrosive environments.

## Figures and Tables

**Table 1:** Bond parameters for Au or Cu wire bonding.

Bond FORCE, grams	Bond Time, ms	Electrical Flame-Off (EFO) Current, mA	EFO Time, ms
30~60	15~10	30~40	700~730

**Table 2:** Corrosion current density and corrosion potential of test specimens by polarization curves.

Specimen	1% NaCl		1% NaCl + 0.01% HCl		1% NaCl + 0.1% HCl		1% NaCl + 1% HCl	
	i_corr_, nA/cm^2^	E_corr_, mV(SCE)	i_corr_, nA/cm^2^	E_corr_, mV(SCE)	i_corr_, nA/cm^2^	E_corr_, mV(SCE)	i_corr_, nA/cm^2^	E_corr_, mV(SCE)
Solid Al	10.67	−911	20.93	−791	76.30	−722	543.00	−754
Sputtered Al	182.00	−1590	69.60	−1240	15.80	−973	72.70	−901
Au wire	0.072	24	0.072	81	0.068	217	0.028	299
Cu wire	2.85	−278	5.31	−293	5.34	−295	8.05	−341

## Data Availability

Data are contained within the article.
